# Repigmentation of Tenacious Vitiligo on Apremilast

**DOI:** 10.1155/2017/2386234

**Published:** 2017-11-06

**Authors:** Sara B. Huff, Lorie D. Gottwald

**Affiliations:** ^1^University of Toledo College of Medicine and Life Sciences, 3000 Arlington Avenue, Toledo, OH 43614, USA; ^2^Department of Dermatology, University of Toledo Medical Center, 3000 Arlington Avenue, Toledo, OH 43614, USA

## Abstract

Vitiligo is a common pigment disorder characterized by acquired loss of function or absence of melanocytes, leading to distinct areas of depigmentation. Physical exam reveals sharply demarcated, depigmented macules or patches on otherwise normal skin. Vitiligo can present at any age, in any skin color. There is no specific serologic marker for diagnosis, but patients often have other autoimmune problems. Treatment options are limited and are difficult given the fact that the pathogenesis of the disease is not well elucidated. We present the case of a 52-year-old woman with vitiligo for over 2 decades. The patient's medical history reveals a lack of response to many different approaches. This case highlights the ability of apremilast, an FDA-approved drug for the treatment of psoriasis and psoriatic arthritis, to achieve repigmentation in a case a vitiligo that has been extremely recalcitrant.

## 1. Introduction

Vitiligo is a common pigment disorder characterized by acquired development of white macules on the skin due to the loss of functioning melanocytes in the skin, the hair, or both [1]. Although the pathogenesis is not known, it is thought to be a cumulative effect of different mechanisms, including autoimmune, neurohormonal, genetic, oxidative stress, and cytotoxic [1–3]. The areas of depigmentation are usually symmetrical, sharply demarcated, and enlarged over time. Vitiligo affects all ages, races, and ethnic groups [2]. Considering the distinct contrast in white patches with the normal skin, vitiligo is more prominent in darker skinned persons. Vitiligo can greatly impact the quality of life for anyone afflicted, no matter what age. Patients report feeling stigmatized, being isolated, and having low self-esteem [1, 4].

Although topical and systemic glucocorticoids, topical calcineurin inhibitors, and narrowband ultraviolet B phototherapy are used to promote repigmentation, they all have varying degrees of success [2, 5, 6]. The response to treatments is lengthy and may be highly different among patients and even inconsistent in different body areas in the same patient. Vitiligo is a chronic disease with a highly unpredictable progression. Studies regarding these treatment options are generally poor or uncontrolled, resulting in the inability to amalgamate conclusions because of the considerable heterogeneity in study design and outcome measure. Hence, alternative and more efficacious therapies are needed for the study and treatment of vitiligo.

## 2. Case Report

**Figure 1:**
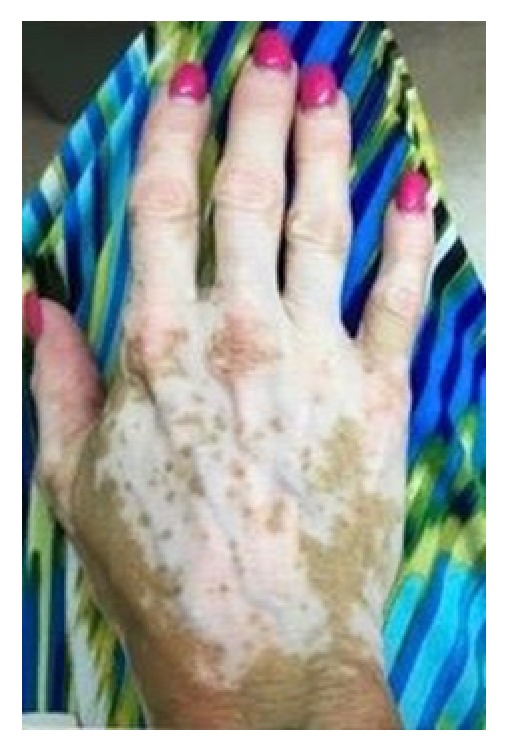
Clinical appearance of the right dorsal hand 5 and a half months after beginning apremilast.

**Figure 2:**
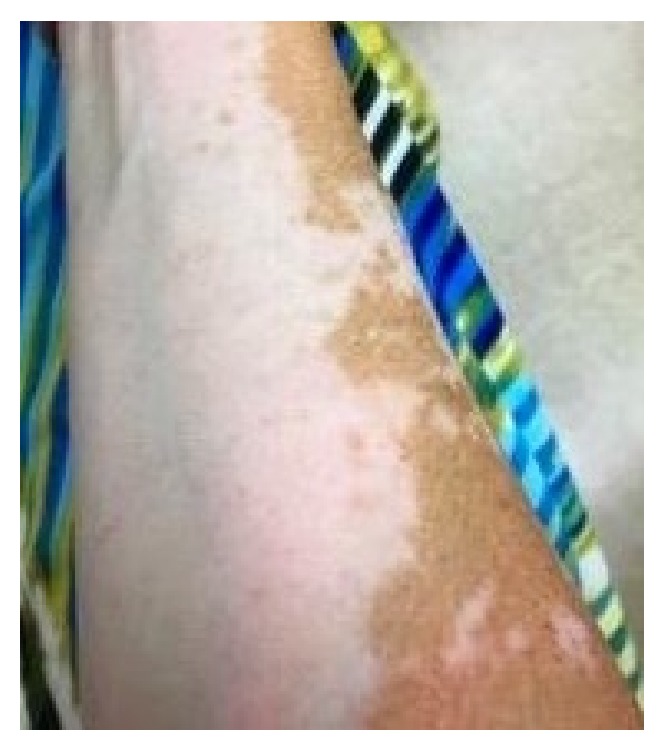
Clinical appearance of the right anterior forearm 5 and a half months after beginning apremilast.

**Figure 3:**
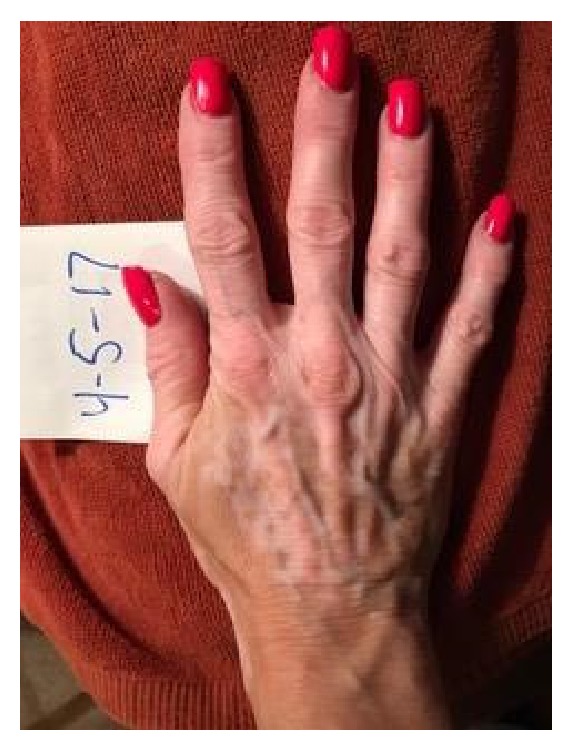
Clinical appearance of the right dorsal hand 13 months after using apremilast 30 mg twice daily.

**Figure 4:**
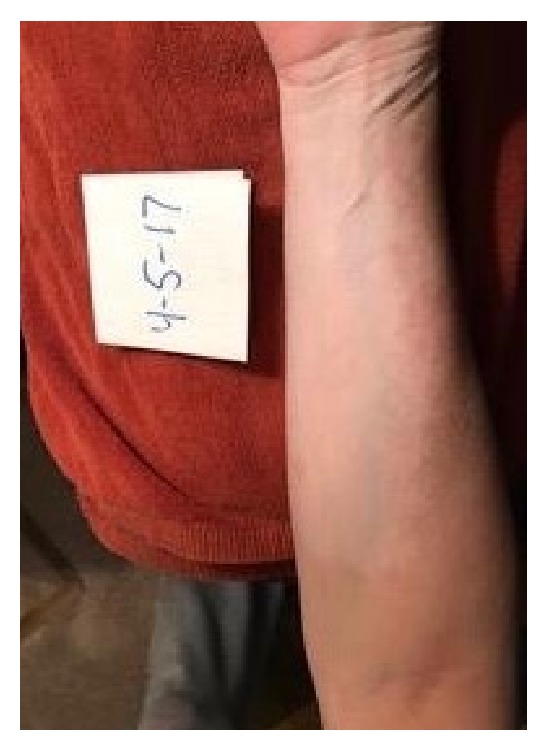
Clinical appearance of the right anterior forearm 13 months after using apremilast 30 mg twice daily.

A 52-year-old woman presented for a follow-up of chronic vitiligo. No other concomitant autoimmune diseases are known in this patient. She developed vitiligo in 1995 and has been seen in this clinic since 1998. She used topical tacrolimus, pimecrolimus, mometasone furoate cream, clobetasol propionate cream, topical psoralen combined with photochemotherapy with UVA, intramuscular triamcinolone acetonide, intramuscular alefacept, subcutaneous etanercept, oral cyclosporine, oral dapsone, and oral prednisone in the past. The patient stopped the use of medications for 5 years prior to re-presenting due to lack of success with repigmentation with the previously listed medications. Her attempt at multiple non-FDA-approved medications illustrates her frustration. At the first visit, the diagnosis of chronic vitiligo was readdressed. The recent data suggesting that apremilast may have a broad effect on inflammation was reviewed and the patient was willing to try apremilast in a non-FDA-approved fashion [7]. After six weeks of apremilast treatment, she reported she believed she was starting to repigment. She continued treatment with apremilast at 30 mg twice daily. Three months after initiating treatment, the patient presented for a follow-up with mild improvement. The patient was bolstered at this time with 60 mg intramuscular triamcinolone acetonide and apremilast 30 mg twice daily was maintained. Note that many years of attempts at steroids as a solo therapy had yielded no improvement. Six weeks following this visit she started to note repigmentation of hands, but felt that normal skin was darkening as well, secondary to ambient sunlight, and she had a bit more pronounced hypopigmentation of perioral area. She received a repeat 60 mg intramuscular triamcinolone acetonide injection and maintained apremilast 30 mg twice daily. Five and a half months after beginning the apremilast treatment, she continued to have areas of improvement, but with a few new areas of hypopigmentation on her feet. The patient felt that repigmentation of hands and forearms was progressing slowly as seen in Figures 1 and 2. At follow-up, 6 and a half months after beginning apremilast treatment, the patient reports repigmentation in bilateral arms, legs, hands, feet, chest, and face. The areas appear red before repigmenting. She had no sun exposure in the interim. At her next follow-up, 8 months after beginning apremilast treatment, the patient experienced fairly significant repigmentation. It was important to continue following the patient without steroids to monitor her progress. At her last visit, 5 months without triamcinolone acetonide supplementation and 11 months after beginning apremilast 30 mg twice daily treatment, the patient was doing extremely well. She is tolerating the apremilast and is repigmenting on her chest and arms 60–70% and now also on her face. The patient provided images of 7 months without triamcinolone acetonide and 13 months after beginning apremilast 30 mg twice daily as shown in Figures 3 and 4. Side effects and rationale regarding apremilast treatment were reviewed at each visit. The patient reported no side effects throughout.

## 3. Discussion

The treatment of vitiligo is challenging given the lack of well controlled studies and inability to pool results based on considerable heterogeneity in study design and outcome measure. Apremilast has been shown to help in autoimmunity from alopecia areata [7]. Our case shows a patient who was treated with apremilast 30 mg twice daily and achieved significant repigmentation in the presence of initial steroid bolstering and continued repigmentation without steroids.

Apremilast is a phosphodiesterase-4 enzyme (PDE-4) inhibitor. Apremilast is FDA-approved for the treatment of patients with moderate to severe plaque psoriasis who are candidates for phototherapy or systemic therapy, as well as for use in psoriatic arthritis. PDE-4 normally degrades cyclic adenosine monophosphate (cAMP) into 5′-adenosine monophosphate. By inhibiting the PDE-4 enzyme specific for cAMP degradation, this results in increased intracellular cAMP levels and thereby regulation of numerous inflammatory mediators through the cAMP second messenger effect (e.g., decreased expression of TNF-*α*, and interleukin- (IL-) 17, IL-23, and interferon gamma, as well as increased IL-10) [8]. When intracellular cAMP is elevated, inflammatory signaling and cytokines are suppressed and anti-inflammatory modulators, such as IL-10, are increased. Vitiligo, like psoriasis, is linked to a dysregulated immune system governed by a proinflammatory cytokine network, involving local and systemic chronic inflammatory processes [8–10]. Melanocytes cultured from vitiligo patient skin samples have been shown to express high levels of cytokines including IL-6 and IL-17 [9]. Positive correlations between levels of IL-17 and disease extent and activity have been found [9]. IL-6 and IL-8 can attract immune components to the skin and may be the link between the triggering event and the initiation of the autoimmune response that results in vitiligo progression [9]. Recent observations have indicated the critical role of altered cellular immunity, autoimmunity, and cytokines in the etiopathogenesis of vitiligo [11]. The use of apremilast, an oral PDE-4 inhibitor for chronic inflammatory diseases, which does not target any single mediator, but rather has a broad range of effects, has been shown to be helpful in the treatment of vitiligo in our patient [8]. There are possible risks to every intervention for vitiligo. Common side effects of apremilast are diarrhea, headache, nausea, vomiting, weight loss, and depression; these adverse events typically present early and are self-limiting. The advantages of using apremilast include ease of oral administration, minimal drug interaction potential, and safety adverse event profile. It should be noted that vitiligo can spontaneously be repigmented. Spontaneous repigmentation would be unlikely in this case, given the chronicity of disease. A PubMed search of articles indexed for MEDLINE using the terms* vitiligo, apremilast, *and* re-pigmentation *revealed that there currently are no known cases of repigmentation in patients with vitiligo on apremilast that have been reported. Our patient presented with chronic vitiligo that was extremely recalcitrant to many different classes of drugs and modalities of delivery.

## 4. Conclusion

Psoriasis, alopecia areata, and vitiligo share a common pathway of autoimmunity, inflammatory signals, and cytokines present, although their pathogenesis is not completely understood. Apremilast is FDA-approved for psoriasis and psoriatic arthritis. Apremilast has also been shown to inhibit the development of alopecia areata [7]. We present this case to demonstrate the ability of apremilast to allow for repigmentation in a patient with chronic recalcitrant vitiligo in conjunction with initial systemic glucocorticoids. More clinical studies, ideally a randomized placebo-controlled trial, would be needed to prove that apremilast leads to repigmentation in vitiligo.

## Supplementary Material

Timeline showing the patient's continual use of apremilast 30 mg BID and when steroid bolstering occured.
