# Supplementary figures and images for: Repigmentation of Tenacious Vitiligo on Apremilast
Source: Case Rep Dermatol Med. 2017 Nov 6;2017:2386234. doi: 10.1155/2017/2386234 (PMC5694993; doi:10.1155/2017/2386234)

## Slide 1
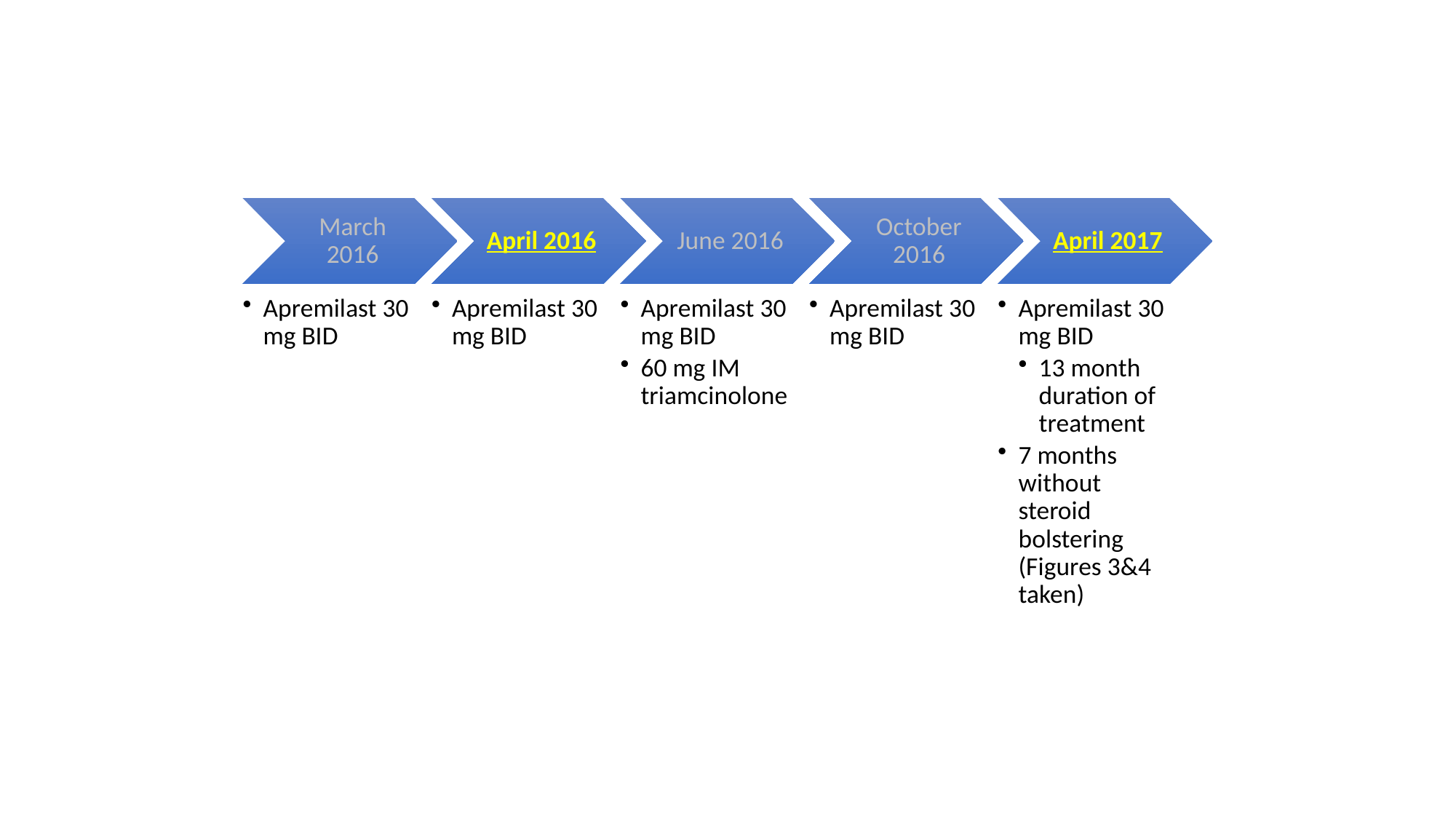

Supplement: Supplementary file 1 — Timeline showing the patient's continual use of apremilast 30 mg BID and when steroid bolstering occured. [file 2386234.f1.pptx]
